# Recent Advances in the Design of Water Based-Flame Retardant Coatings for Polyester and Polyester-Cotton Blends

**DOI:** 10.3390/polym8100357

**Published:** 2016-10-11

**Authors:** Jenny Alongi, Federico Carosio, Paul Kiekens

**Affiliations:** 1 Department of Chemistry, University of Milan, Milano 20133, Italy; 2 Dipartimento di Scienza Applicata e Tecnologia, Politecnico di Torino, Alessandria Campus, Alessandria 15121, Italy; federico.carosio@polito.it; 3 Department of Textiles, University of Ghent, Gent 9052, Belgium; paul.kiekens@ugent.be

**Keywords:** nanoparticle adsorption, layer by layer, flame retardancy, polyester, polyester-cotton blend

## Abstract

Over the last ten years a new trend of research activities regarding the flame retardancy of polymeric materials has arisen. Indeed, the continuous search for new flame retardant systems able to replace the traditional approaches has encouraged alternative solutions, mainly centred on nanotechnology. In this context, the deposition of nanostructured coatings on fabrics appears to be the most appealing and performance suitable approach. To this aim, different strategies can be exploited: from the deposition of a single monolayer consisting of inorganic nanoparticles (single-step adsorption) to the building-up of more complex architectures derived from layer by layer assembly (multi-step adsorption). The present paper aims to review the application of such systems in the field of polyester and polyester-cotton blend fabrics. The results collated by the authors are discussed and compared with those published in the literature on the basis of the different deposition methods adopted. A critical analysis of the advantages and disadvantages exhibited by these approaches is also presented.

## 1. Introduction

As is well known, polyester is one of the commonest synthetic fibres used in the textile field for apparel, home furnishing, and auto upholstery thanks to its features (e.g., resistance to stretching, shrinkage, abrasion, wrinkling, resistance to most chemicals, easily washed and quick drying) [1].

First introduced as Dacron by DuPont in 1951, it readily replaced nylon in a number of applications; in doing so, polyester became the most used fibre (38%) after cotton (47%) in 2000, and far ahead of other synthetic polymers in terms of both production and consumption volume/value [2].

Polyethylene terephthalate (PET) or polyester, as commonly defined in textile, is synthesised by condensation polymerisation of ethylene glycol and terephthalic acid. Subsequently, it can be transformed by melt compounding/extrusion, spinning and drawing. Its popularity largely derived from its easy-care characteristics, long durability and high compatibility with cotton in blends. Additional qualities are represented by its very low moisture absorbency (five times lower than cotton) and good mechanical properties: medium or high tenacity polyester fibres have E moduli that range from 10.6 to 13.2 N/tex (deformation at break 7%–15%) while cotton modulus ranges from 3.9 to 7.3 N/tex (deformation at break 5.6%–7.1%) [3,4].

Many manufacturers across the world produce polyester under different commercial names with almost tailor-made properties [2]. However, as for all other polymers, one of the biggest drawbacks of polyester is represented by its flammability and tendency to burn with the formation of molten droplets that can easily spread the fire to other materials. It is thus necessary to improve the fire behaviour of polyester making it flame retarded.

In order to do this, three approaches can be exploited:

- use of flame retardant (FR) comonomers during copolymerization,
- introduction of an FR additive during extrusion,
- application of FR finishes or coatings [5,6].

The first two methods give inherently flame-retardant polyester fibres. The most famous products currently targets of the market belong to these methods and are Trevira^®^ CS and Trevira^®^ FR, both manufactured by copolymerizing a bifunctional organophosphorus compound based on a phosphinic acid derivative. The LOI, Limiting Oxygen Index, (loss on ignition) of Trevira^®^ CS fabric, having 0.6% phosphorus, is 28% and the burning fabric does not give rise to burning molten droplets. In addition, the production of flame retardant Trevira^®^ has been found to be a clean process compared to others for artificial and natural fabrics (e.g., cotton). Tests on the toxicity of burning fabrics at increasing temperatures showed that Trevira^®^ CS and FR polyesters are even less toxic than FR-treated cottons. Indeed, fumes released from Trevira^®^ CS polyester at 700 °C indicate a mortality rate of only 8% in experimental animal tests compared to 83% for FR-cotton. Thus, Trevira^®^ CS and FR have been certified for the Oeko-Tex 100 standard [5].

Other examples of comonomers employed for making polyester flame retardant are sulphone–phosphonate copolymers manufactured by Toyobo GH and a spirocyclic pentaerythritol di(phosphate acid monochloride) synthesized by Ma et al. [7].

As mentioned above, the alternative approach for flame retarded polyesters is the application of finishes or coatings. These systems should promote char formation by reducing the thermoplasticity [5]. Day et al. [8,9] investigated the flammable behaviour of polyester fibre by using a series of phosphorus- and bromine-containing FRs as finishing agents. Alternatives to tris(2,3-dibromopropyl) phosphate, a known carcinogen species, were applied from tetrahydrofuran solution. The chemicals used were tris(2,3-dibromo-2-methylpropyl)phosphate, tris(2,3-dibromo-3,3-dimethylpropyl)phosphate, etc. The pyrolysis and combustion species of so-treated PET showed significant release of HBr, which is capable of completely inhibiting the combustion reactions occurring in the gas phase. The authors noted also the formation of P-based species able to act in the condensed phase [8]. Alternative systems consisting of low melting solid or liquid brominated compounds in combination with colloidal antimony oxide, tetraphenoxydiaminocyclotriphosphazene dispersions and hexabromocyclododecane have been proposed, as well [5]. In particular, the former approach turned out to be extremely durable being resistant to multiple launderings and dry cleanings.

Another approach is represented by the thermosol processes, which involve the aqueous padding of the FR, followed by drying at high temperatures (190–210 °C) for short times (30–40 s) in order to let FR migrate into the fibers. Rhodia’s Antiblaze^®^ 19 or Amgard CU^®^ has been used as a major product in such treatments [10].

Although real and tangible solutions have been found for polyester (i.e., Trevira^®^ CS and FR), the same results have not been achieved for its blends with cotton, notwithstanding the great benefits represented by the use of polyester-cotton blends. Indeed, flame retardation of these systems is still a complex and unsolved problem due to the differential thermal and flame behaviour of polyester and cotton. Most of the approaches documented in the literature [11,12,13] exhibit limitations due to their toxicity, or difficulty in maintaining desirable aesthetic and technical performances. Miller et al. [11] observed that a specific polyester-cotton blend (approximately 30 wt % cotton content) ignites sooner, burns faster, and releases more volatile hydrocarbons than those produced by either polyester or cotton. Alongi et al. [14,15] have achieved analogous results when polyester-cotton blends having 15 or 35 wt % of cellulosic counterpart are exposed to a 35 kW/m^2^ heat flux in a cone calorimeter. On ignition, cellulose forms char, providing a scaffold for the molten polyester and preventing its escape from the flaming zone. Miller et al. thought that the interaction could be of a chemical nature (based on interactions of pyrolysis products in the gas phase) or of physical origin, which alters the heat, oxygen and mass transfer.

FRs containing phosphorus, nitrogen, and antimony/halogen compounds have proven to be very efficient for such blends acting in both the condensed and vapour phases [16,17]; in spite of these results, however, the research on new and better performing solutions is still open.

**Figure 1:**
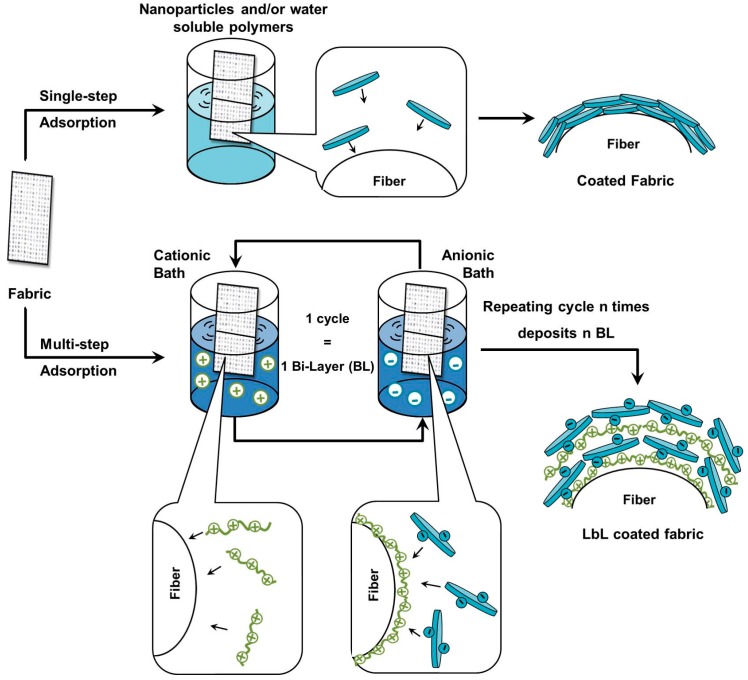
Schematic representation of single- and multi-step deposition techniques presented in this review.

Recent concerns about the toxicity and sustainability of commonly used FRs have opened the way for the development of greener and efficient solutions. In this scenario, nanostructured coatings have attracted the interest of the scientific community [18,19,20]. Among “old” and “new” coating deposition techniques, the nanoparticle (NP) adsorption [21,22] and the layer by layer (LbL) assembly [23,24,25,26,27,28,29,30,31] have been studied and reinvented to match the FR purpose. Both techniques are very simple and straightforward as they rely on the single-step or multi-step surface adsorption of nanoparticles or polyelectrolytes from diluted (≤1 wt %) water-based suspensions or solutions, as shown in Figure 1.

While both solutions present advantages such as simplicity and “green” nature (the solvent is water, the process occurs at room conditions and it is possible to recycle the deposition baths after use), the multi-step adsorption achieved by means of the LbL technique offers additional benefits. Indeed, as the LbL allows for the build up of stratified coatings relying on electrostatic interactions occurring between oppositely charged polyelectrolytes or nanoparticles, the components can be selected among a wide range of reagents thus giving access to nearly infinite possibilities. Beside this, the outcome of the LbL can be highly affected by the deposition parameters (such as pH, ionic strength, temperature) further increasing the versatility of the process.

In recent years, early works employing the two techniques provided encouraging results for both polyester and polyester-cotton blends. For example, the single-step adsorption of lamellar nanoparticles was found able to remarkably increase (+103%) the time required to ignite polyester samples during cone calorimetry tests. Similarly impressive results have been obtained by the multi-step deposition of silica nanoparticles: treated polyester fabrics showed self-extinguishing properties and suppressed melt dripping during vertical flame spread tests.

The key factor in the success of such approaches has been found in their capability to act as char-promoting agents.

Indeed, completely inorganic architectures consisting of a monolayer deposited by NP adsorption and intumescent assemblies enriched by inorganic NP assembled by LbL turned out to act as a thermal insulator barrier able to reduce the heat, oxygen and mass transfer from the atmosphere and surrounding substrate. In doing so, the polymer tends to undergo pyrolysis towards char formation rather than release of volatile gases that sustain combustion.

Since the first papers were published, many others, mainly from our research group, followed. Hereafter, a brief overview of the results collected over the last 10 years by the same authors on the application of NP adsorption and LbL assembly for polyester and polyester-cotton blends is reported and discussed in comparison with homologous systems present in the literature. In addition, a critical discussion about advantages and disadvantages of such approaches is proposed.

## 2. Surface Key Role in Combustion

As mentioned above, nanostructured coatings exhibiting peculiar flame retardant features can be deposited by either single-step or multi-step adsorption on different surface substrates. In doing so, the combustion of the underlying polymer can be drastically modified as the surface can play a predominant role, in particular in the pre-ignition and ignition phases. These architectures are able to favour degradation paths that lead to the production of carbonaceous residues rather than combustible volatile gasses as they can either reradiate heat or slow down heat transmission and volatile diffusion, without affecting polymer bulk properties.

Indeed, being the interface between gas and condensed phase, the surface represents the region that controls mass and heat transfers that are the processes responsible for flame fuelling [23]. The heat (from a flame or heat source) reaching the polymer is transmitted via its surface to the bulk; at the same time, volatile products of thermal degradation diffuse from the bulk and across the surface towards the gas phase, feeding the flame. It is then easy to visualize how the surface can play such a key role in polymer ignition and combustion. Indeed, one of the most valuable fire retardant strategies pursued by bulk addition relies on the production or accumulation of a thermally stable surface layer able to act as a barrier to mass and/or heat exchange. This layer, often consisting of inorganic nanoparticles, is built after ignition as a consequence of the decomposition of the polymer surface layer.

However, since time is required to achieve an efficient protection, the performances of the bulk approach are limited or totally non-existent during the pre-ignition and ignition phases. On the other hand, by building up a pre-existent fire protection onto the original polymer surface (as schematically depicted in Figure 1), its effectiveness will be larger.

Here, it is shown how the combination of the above mentioned concepts and deposition techniques have been successfully exploited for the surface protection of polyester and polyester-cotton blends. Inorganic coatings consisting of mainly ceramic particles and silicates have proven to be efficient FRs acting as physical barriers, regardless of the deposition method. In the case of inorganic-organic (mostly intumescent) systems, the joint action between the physical barrier exerted by the inorganic counterpart and the char-forming action exerted by the organic part turned out to be successful. Under the following headings, these aspects will be detailed and discussed.

### 2.1. Single-Step Adsorption

#### 2.1.1. Polyester

**Figure 2:**
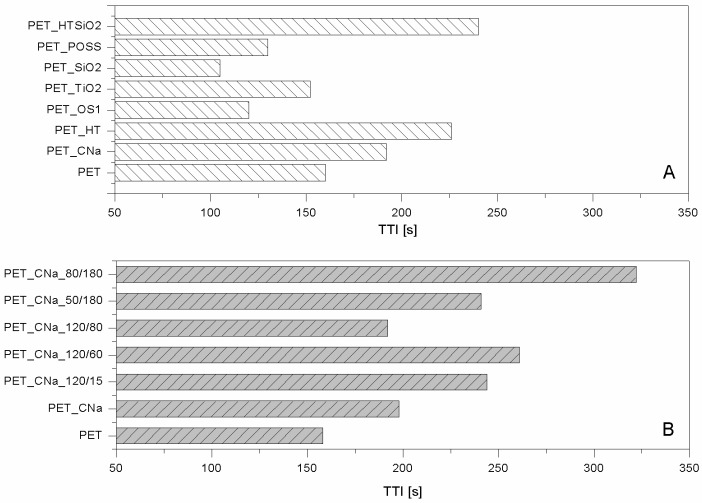
TTI values of nanoparticle (NP)-treated polyethylene terephthalate (PET) fabrics (**A**) and treated initially by plasma and then with CNa suspension (**B**) [33,35]. Data obtained by cone calorimetry at 35 kW/m^2^.

**Figure 3:**
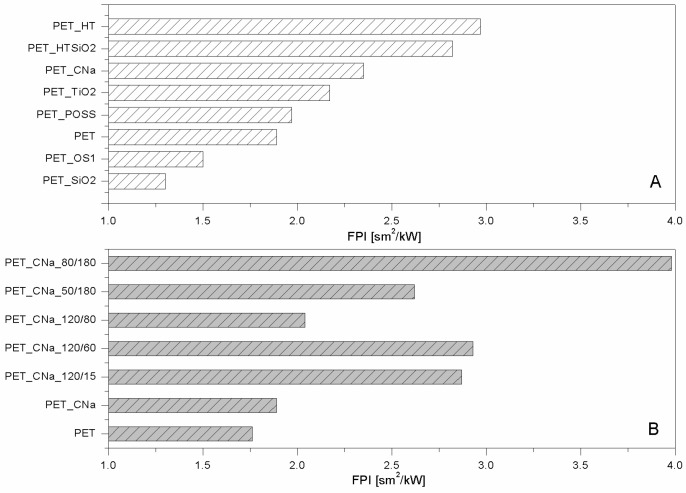
FPI values of NP-treated PET fabrics (**A**) and treated initially by plasma and then with CNa suspension (**B**) [33,35]. Data obtained by cone calorimetry at 35 kW/m^2^.

As far as polyester fabrics are concerned, different NPs having either lamellar (e.g., montmorillonites (CNa), hydrotalcite (HT), and bohemite (OS)) or globular (silica, SiO_2_), titania (TiO_2_) and octapropylammonium polyhedral oligomeric silsesquioxane—POSS^®^) shape were studied as potential FRs. The resulting properties were assessed by cone calorimetry under a 35 kW/m^2^ heat flux. The collected data are summarised in Table 1. CNa significantly increased PET Time to Ignition, TTI (192 vs. 160 s, respectively for PET_CNa vs. PET, see Figure 2A) and reduced both Effective of Heat Combustion (EHC, 17.5 vs. 21.8 MJ/kg) and Heat Release Rate peak (PHRR, 80 vs. 90 kW/m^2^) [22]. As a consequence, Fire Performance Index (FPI) value of PET_CNa is higher than that of untreated PET (see Figure 3A). This finding suggests that the PET combustion mechanism has been partially modified by the presence of a CNa monolayer deposited on the fabrics; indeed, such clay nanocoating acting as an insulating ceramic barrier somehow protected the polyester from heat, oxygen and mass transfer during combustion. Consequently, the ignition was postponed and the effective heat reduced as a lower amount of polymer burns. In addition, its presence caused a slight decrease of the CO and CO_2_ yields. The Total Heat Release (THR) is almost constant within the experimental error.

The results already discussed have encouraged the pursuance of the approach of NP adsorption (and in particular the use of CNa due to its favourable aspect ratio and swelling properties in water), but have also highlighted the limitations derived from a deposition consisting of a single immersion step, in particular for non-hydrophilic substrates like polyester. Indeed, observing the morphology of CNa-treated fabrics (scanning electron microscopy—SEM—micrographs reported in ref. [35]), it has been found that NPs do not completely and homogeneously cover PET fibres; as a consequence, the resulting flame retardant performances turned out to be strongly limited. Thus, in order to further increase the number of PET/CNa interactions, a pre-treatment etching of the fabric by cold oxygen plasma was carried out under different experimental conditions of power (50, 80, and 120 W) and time (15, 60, 80 and 180 s).

The results collected by cone calorimetry reported in Table 2 show how much the plasma pre-treatment can improve CNa performances, by increasing both clay distribution densities on the surface and PET/CNa interactions. More specifically, the higher number of deposited NPs resulted in a drastic effect on PET TTI delay (see Figure 2B), regardless of treatment power or time; despite this, the combustion kinetic parameters were not significantly affected (e.g., PHRR in Table 2). An important aspect to highlight is the trend of TTI increase as a function of plasma conditions. At the highest power (namely, 120 w), 60 s of treatment gave the highest TTI increase (almost comparable with that of 15 s): longer treatment does not further increase PET TTI.

The sample exhibiting the highest performances was prepared by pre-treating PET fabrics at 80 w for 180 s; this sample displayed an increase of TTI up to 104% (from 158 to 322 s for PET and PET_CNa_80/180, respectively, Figure 2B) and a small reduction of PHRR (10%); as a consequence, such a sample yielded the highest FPI value (Table 2 and Figure 3B). It is also interesting to observe the FPI trend as a function of plasma power and time, reported in Figure 3B.

CNa performances have been compared with those of other two lamellar NPs, namely, a carbonate hydrotalcite and a p-toluenesulphonate bohemite.

Hydrotalcites are well known anionic nanoclays, having a chemical formula of Mg_6_Al_2_(CO_3_)(OH_16_)·4(H_2_O) and organized as layered double hydroxides [36]. The high water content present in their structure makes them potential flame retardant systems able to protect a polymer, as they form a ceramic layer during combustion, releasing a great amount of water upon heating. In this way, the degradation products released by the polymer are diluted, favouring a remarkable delay of its ignition and/or a reduction of combustion kinetics. For these reasons, PET fabrics were treated with a carbonate HT solution and subjected to cone calorimetry tests (see Table 1) [22]. In detail, HT is responsible for PET TTI increase from 166 up to 226 s (Figure 2A) and PHRR decrease from 90 to 56 kW/m^2^. Thus, FPI of PET_HT is drastically higher than that of untreated PET and also of PET_CNa (Table 1 and Figure 3B). This difference between these two values easily finds an explanation, comparing the TTI values of the two samples (192 vs. 226 s for PET_CNa and PET_HT, respectively). The presence of a higher water amount in the HT structure with respect to that internal in CNa galleries has a stronger effect in diluting the volatiles released by PET degradation before its ignition.

The third lamellar NP employed for treating PET fabrics is a p-toluenesulphonate bohemite. Bohemites theoretically have intrinsic flame retardant features as they are aluminium oxide hydroxides, γ-AlO(OH), that dehydrate in the range of 100–300 °C, releasing water and subsequently transforming into crystalline γ-Al_2_O_3_ phase at circa 420 °C [36]. Similarly to HT, also bohemites should promote a strong diluting effect on the volatile products generated by polymers upon heating hence polymer ignition should be delayed. In addition, the ceramic barrier resulting from the presence of the just-formed alumina inhibits further combustion. Unfortunately, in spite of these considerations, when polyester fabrics are treated with such NP, PET TTI does not change in a significant way (Table 1 and Figure 2A); however, a reduction in EHC (15.0 vs. 15.8 MJ/kg for PET_OS1 and PET, respectively, Table 1), PHRR (from 90 to 70 kW/m^2^) and CO and CO_2_ yields was registered.

Comparing the lamellar nanoparticles in terms of TTI (Figure 1A) and FPI (Figure 3A) improvements, the highest performances were achieved by treating PET with HT. On the other hand, including in this discussion a possible etching pre-treatment of fabrics, CNa exhibited the highest performances (namely, PET_CNa_80/180 sample).

As mentioned above, globular nanoparticles such as titania, silica, and octapropylammonium POSS have been taken into consideration as flame retardant systems for polyester, as well [32]. In particular, these nanoparticles acting as smoke suppressants reduced the CO and CO_2_ yields in a remarkable way (Table 1). Unfortunately, silica particles did not impart good flame retardancy to polyester, as well visible comparing the corresponding FPI values (Figure 3A). On the contrary, among the globular NPs, POSS exhibited the best performances by increasing PET TTI (Figure 2A) and significantly reducing its PHRR as well as CO and CO_2_ release.

These results together with the morphologies reported in ref. [32] pointed out the presence of a low amount of titania and silica deposited on the fibre surfaces similarly to that observed for CNa. For this reason, an efficient physical barrier capable of protecting the substrate from fire was not achieved.

In order to overcome such a problem, the combination of lamellar and globular nanoparticles was proposed for reaching the optimal flame retardant performances of polyester [32]. Thus, the features of HT and silica were combined in treating PET. To this aim, PET was initially treated with an HT suspension and then with a silica one; in doing so, two consequent layers were deposited on the PET fabrics. It is important to highlight that this approach somehow mimics what occurs in a LbL assembly and thus can be considered a sort of precursor of it. The results are the best collected for PET by using the approach of NP adsorption, as evidenced observing the TTI increase (Table 1 and Figure 2A) and FPI value (Figure 3A). In addition, further analyses have shown that this system is able to reduce the production of CO and CO_2_ in a remarkable way (Table 3).

Beside the use of nanoparticles, water-soluble polymers or monomers to be polymerized in situ can be employed for the production of a fire protective treatment. In this approach an FR additive or FR monomer is employed in order to impart the final FR properties to the deposited coating. The main action of these coatings is similar to that of NPs: upon flame exposure the coating can swell into an expanded protective layer that limits heat transmission and volatiles release. The degree of expansion and the mechanism for barrier formation depend on the chemistry of the system.

Starch has been used as continuous phase in combination with sodium polyborate (SPB) evaluating different weight ratios between the polysaccharide and the FR [37]. PET non-woven treated with the best formulation, achieved at a 0.134 ratio with a total add on of 48.4 wt %, and was found capable of enduring intensive heating by a premixed gas burner for 12 min. Table 3 reports the performances obtained at different coating compositions.

The good performances achieved by the starch/SPB mixture were justified as follows: upon exposure to heat, SPB forms a foamed structure that insulates starch and improves its charring reactions, the release of water from starch enhances the foaming of SPB and dilutes the decomposition products, the SPB foam and starch carbonaceous residue insulate the substrate from heat and oxygen thus resulting in an FR action.

In another application PET fabrics were coated by polyaniline synthesized via in situ chemical polymerization of aniline in the presence of ammonium persulfate (as the oxidant), HCl, and H_3_PO_4_ (as the dopant) [38]. The coating composition suggests intumescent formulation, the phosphoric acid works as acid source while the polyaniline represents both nitrogen and carbon sources. The effects of different oxidant to monomer ratios on the achieved FR properties were evaluated by LOI. Fabrics treated with a 1:2 molar ratio of ammonium persulfate to aniline resulting in the highest LOI (42%).

Recently, Luo et al. combined the two approaches described above by preparing a FR polyurethane (PU) waterborne resin, that was further, added with a commercial FR in order to exploit the synergistic effect of P–N systems to reduce the formation of flammable volatiles as well as catalyse char formation [39]. In details, PET fabrics were coated with a waterborne polyurethane latex copolymerized with either Fyrol-6 or octahydro-2,7-di(*N*,*N*-dimethylamino)-1,6,3,8,2,7-dioxadiazadiphosphecine (ODDP) or the combination of both including Exolit^®^ OP550 in the final suspension. Regardless of the adopted formulation, treated PET fabrics (coating add-on was kept constant at 19 wt %) exhibited self-extinguishing behaviour in both horizontal and vertical flammability tests (UL 94 rating) as well as LOI values between ca. 25% and 27%. Table 4 shows the detailed results.

With respect to nanoparticle adsorption, the use of a water-soluble polymer in combination with FR may offer different advantages such as better surface coverage, the possibility to include different FR additives and multiple crosslinking strategies for improving durability. On the other hand, the coating add-on is normally higher than NP adsorption with possible detrimental effects on the physical and comfort properties of the treated fabric.

#### 2.1.2. Polyester-Cotton Blends

**Figure 4:**
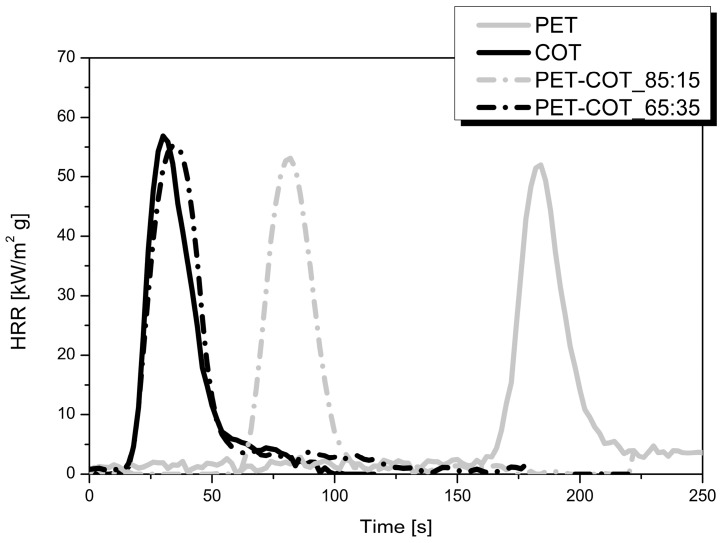
Heat Release Rate (HRR) curves of PET, COT, PET-COT_85:15 PET-COT_65:35 fabrics. Data obtained by cone calorimetry under 35 kW/m^2^.

As mentioned in the Introduction, one of the biggest challenges for industrial applications is replacing PET with some blends containing cotton fibres. In this scenario, we investigated the NP adsorption approach in order to find the best nanoparticles able to protect two polyester-cotton (PET-COT) blends having 15 and 35 wt % of cotton, respectively. To this aim, we initially studied how such materials react to a 35 kW/m^2^ heat flux to understand whether their behaviour was more similar to that of PET or cotton. This influenced the choice of NPs to employ. Figure 4 reports the HRR curves for untreated PET, cotton, and two blends under investigation. It is clear that, regardless of the cotton amount, the TTIs of the two blends are more similar to those of cotton as compared with PET, indicating that cotton behaviour is dominating in the pre-ignition phase. Thus, on the basis of the results already collected for cotton [22], CNa, HT and silica were selected.

**Figure 5:**
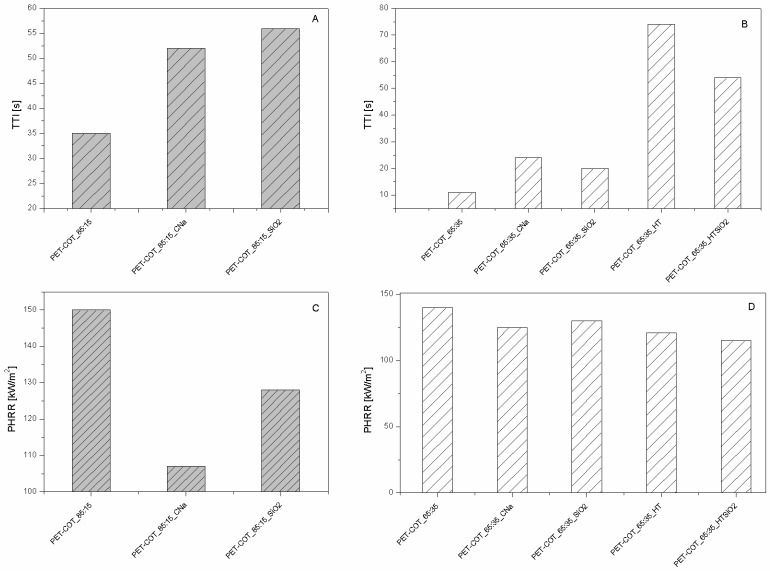
TTI and PHRR values of NP-treated PET-COT_85:15 (**A**,**B**, respectively) and NP-treated PET-COT_65:35 fabrics (**C**,**D**, respectively). Data obtained by cone calorimetry at 35 kW/m^2^.

As far as CNa is concerned (Table 5 and Table 6), both blends exhibited a higher TTI (Figure 5A,B) and a lower PHRR (Figure 5C,D) due to the presence of NP. Referring to silica, the NP effect is comparable for the two substrates; no significant differences in performances were observed, also in terms of FPI value improvements (see Figure 5). For the blend having a higher cotton amount, HT was tested as well, since as described before, alone or in combination with silica it exhibited good performances for both PET and cotton fabrics [22]. In such cases, the best performances were achieved for the blend treated with one layer of HT, even though significant improvements were achieved also by coupling HT with silica. The latter results highlight how the subsequent adsorption of oppositely charged NPs in a layer by layer fashion can be the right route for the assembly of a performing flame retardant nanocoating.

**Figure 6:**
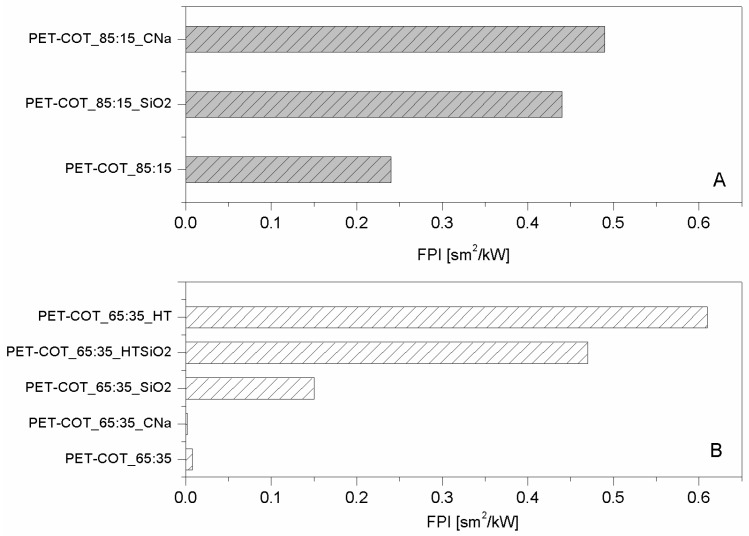
FPI values of NP-treated PET-COT_85:15 (**A**) and NP-treated PET-COT_65:35 fabrics (**B**). Data obtained by cone calorimetry at 35 kW/m^2^.

**Figure 7:**
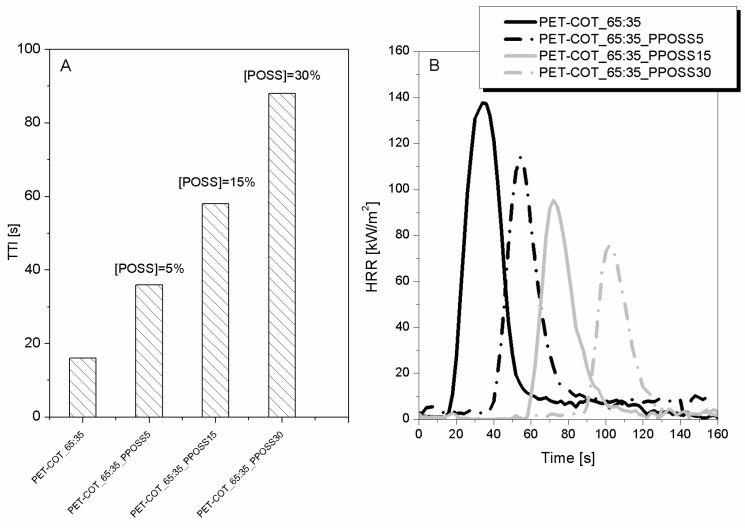
TTI values (**A**) and HRR curves (**B**) of PET-COT_65:35 fabrics treated with different POSS amounts and a phosphorus-based FR. Data obtained by cone calorimetry at 35 kW/m^2^.

For a possible industrial application, POSS has proven to be the most promising NP to improve PET flame retardancy. Indeed, following the ISO15025 standard, these samples did not burn when exposed to a 2.5 cm methane flame. Unfortunately, the same trend was not observed for a PET-COT blend. Thus, combination of a phosphorus-based flame retardant (namely, Pyrovatex^®^-like [6]) with POSS was experimented in order to enhance the overall flame retardancy level of such blends. Figure 6 shows the collected results by cone calorimetry for PET-COT_65:35 treated with Pyrovatex^®^ at 10 wt % add-on (the add-on normally employed is 19 wt %) and different POSS amounts (namely, 5, 15, and 30 wt %). By increasing the POSS amount, TTI increases (up to 500% improvement) and PHRR decreases (up to 50% reduction) in a remarkable way (Figure 7A,B, respectively).

### 2.2. LbL Assembly

In the following section, the results collected exploiting LbL assembly on polyester and polyester-cotton blends are thoroughly described. As depicted in Figure 1, the LbL assembly represents the evolution of single-step adsorption processes. The possibility to employ different components in each bath and their intimate mixture within the coating represent the key points of this technique. As described in the following chapters, researchers have started from relatively simple architectures, inspired from the single-step processes, eventually depositing complex architectures with hybrid intumescent/inorganic FR actions.

#### 2.2.1. Polyester

As mentioned above, a monolayer of silica did not significantly modify PET combustion, exerting an efficient protection (Figure 2A and Figure 3A, Table 1); indeed, apart from a small reduction of EHC, the other combustion parameters assessed by cone calorimetry (e.g., TTI and PHRR) remain almost constant. In spite of this, the EHC reduction shows real proof of silica potentialities. Indeed, it is likely that silica may exert good protection but only when it homogeneously covers the fibres. Such hypothesis is supported by the results collected in other studies where silica based nanocoatings were deposited via sol-gel processes [14,40].

**Figure 8:**
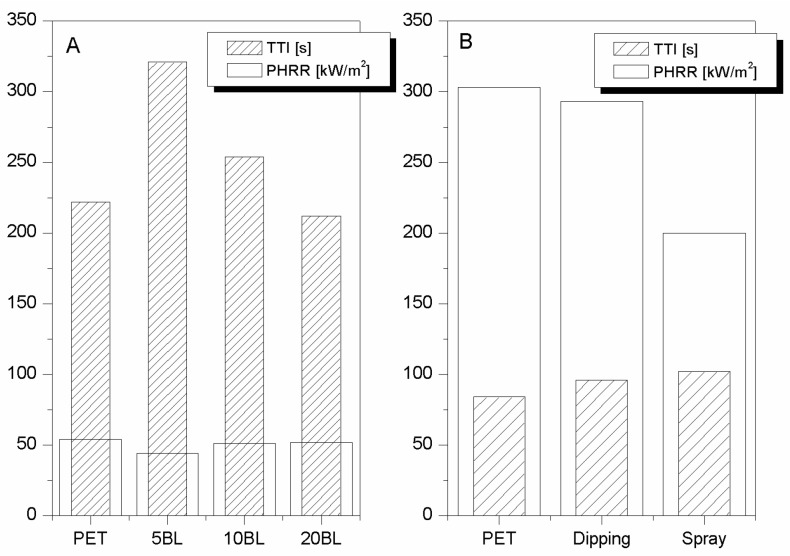
TTI and PHRR values of 171 g/m^2^ PET fabrics treated with 5, 10, and 20 BL of silica/silica nanoparticles deposited by dipping (**A**) [25]. Comparison between same LbL architectures deposited by dipping and spray on 490 g/m^2^ PET (**B**) [26]. Data obtained by cone calorimetry at 35 kW/m^2^.

**Figure 9:**
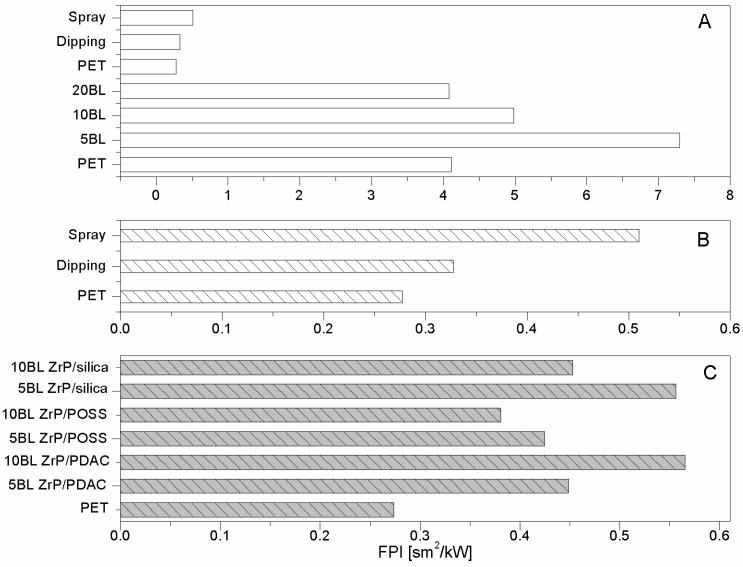
FPI values of untreated and LbL-treated PET fabrics (**A**: ref. [25], **B**: [26] and **C**: [27]). Data obtained by cone calorimetry at 35 kW/m^2^.

Therefore, the possibility to better assemble silica nanoparticles through a LbL strategy was attempted. To this aim 5, 10, and 20 BL (bilayer) of oppositely charged silica particles were deposited on PET fabrics (area density: 171 g/m^2^) [25]; the collected data are summarised in Table 7 and some of which (namely, TTI and PHRR) is plotted in Figure 8A. It is clear that 5 and 10 BL increase PET TTI whereas 20 BL have no effect. On the other hand, only 5 BL are able to significantly reduce PET PHRR. As a result, 5 BL show a higher FPI value than those of untreated, 10 and 20 BL-treated fabrics (Figure 9A). Moreover, as reported in Table 8, such samples also showed the best performances in protecting PET from the application of a methane flame during the vertical strip test [25]. It was observed that, by increasing the BL number, some zones of the fibres were not completely covered by the coating and this morphology was ascribed to assembly collapse.

Although dipping is one of the most common and simplest methods for assembling LbL coatings, spray may be more advantageous for its efficiency and feasibility on an industrial scale, as reviewed by Schaaf et al. [41]. As an example, spray-assisted silica-based LbL architectures were exploited for making flame retardant cotton: more specifically, among the different methods investigated (dipping, vertical and horizontal spray), the most homogeneous and consistent coatings were deposited by using a horizontal spray, which has proven to confer the highest flame retardancy performances.

Thus, spray has been exploited also for PET and compared with dipping. To this aim, the same architecture consisting of 5 silica/silica BL was deposited on high-density PET fabrics (490 g/m^2^) [26]. Figure 8B and Figure 9B show that horizontal spray is more effective than dipping to create a flame retardant LbL coating (−34% and 3% PHRR reduction, respectively, Table 7). This difference depends on the different LbL growth imparted by the two methods under investigation and PET area density. Indeed, as it is well known, spray allows for a homogeneous coverage of the surface from early BL; conversely, by dipping coating growth occurs following an island-growth pattern. Thus, sometimes it is possible to have some zones not completely protected by the coating if the number of deposition steps is not high enough. Analogous results were observed for cotton fabrics [42].

**Figure 10:**
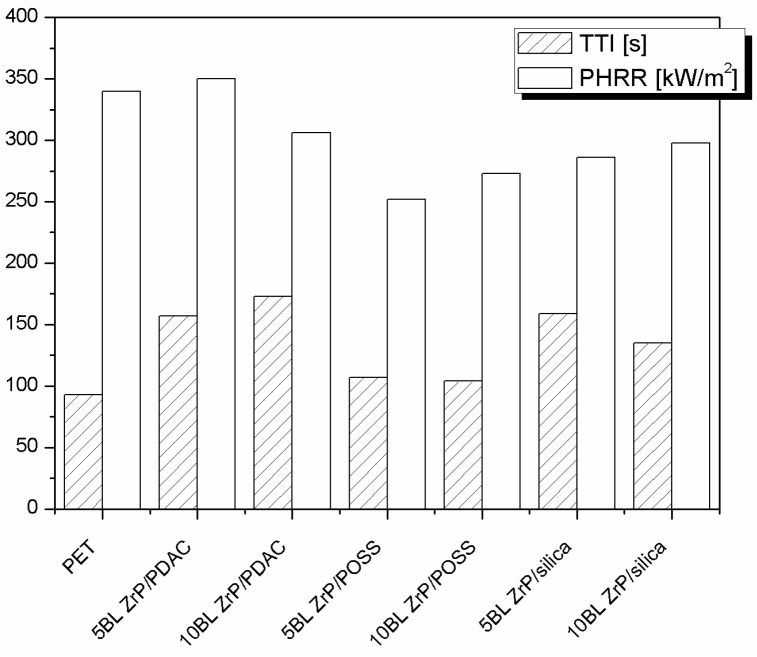
TTI and PHRR values of PET fabrics treated with 5 and 10 BL of ZrP coupled with PDAC, POSS or silica [25]. Data obtained by cone calorimetry under 35 kW/m^2^.

Pursuing the research, it became clear that it was necessary to change the perspective for further improving the performances of LbL architectures for PET. Indeed, although the ceramic barrier created by inorganic nanoparticles had some encouraging results, the final properties were not completely satisfying. Therefore, the research was addressed towards the design of new architectures consisting of both inorganic globular nanoparticles and species having intrinsic flame retardant features such as ammonium polyphosphate (APP) [43] or zirconium phosphate (ZrP) nanoplateletes [44]. The latter species was chosen as the negative counterpart in LBL assemblies coupled with a polymeric polyelectrolyte (namely, polydiallyldimethylammonium chloride, PDAC) and two inorganic nanoparticles (POSS and silica) [27]. So-treated PET exhibited a general increase of TTI and PHRR reduction (see Table 7 and Figure 10). More specifically, 10 BL ZrP/PDAC exhibited the highest TTI while 5 BL ZrP/ POSS the lowest PHRR. Calculating the FPI values for each sample and plotting them (see Figure 9C), it is possible to establish that the highest performances were however achieved by both 10 BL ZrP/PDAC and 5 BL ZrP/silica whose FPI values are comparable, although the latter sample can be considered the best, as it increases PET TTI from 93 to 159 s and reduces its PHRR by 20%. Comparing the performances of such samples (Figure 9C) with those of PET treated with 5 silica/silica BL (namely, dipping in Figure 9B) [25], it is clear that 5 BL ZrP/silica has superior flame retardant properties and thus the joint action between ZrP and silica represents the right route to follow.

Other research groups have also pursued research on nanoparticle-based LbL coatings. Dasari et al. investigated the effects of a branched polyethylenimine (BPEI)/CNa coating on the flame retardancy and physiological comfort of PET fabrics [45]. The presence of the coating was found to impart an overall improvement to the comfort of the fabric due to an increased hydrophilicity; on the other hand, air permeability and water transport capacity were reduced due to the well-known barrier properties of BPEI/CNa LbL coatings. The achieved FR properties were evaluated by means of pyrolysis-combustion flow calorimetry (PCFC); the deposition of the coating reduced the peak of heat release rate and slightly influenced the total heat release as a function of BL number. Beside flammability, preliminary durability tests were then carried out; Table 9 summarizes the achieved results.

Recently, similar architectures were deposited employing chitosan (Chi) as positive counterpart and CNa or titanate nanotubes (TNTs) in BL coatings or with both nanoparticles in more complex architectures consisting of four layers of repetitive units (Chi/CNa/Chi/TNT, quadlayer, QL) [46]. Coatings containing CNa nanoparticles gave the best results in terms of melt dripping suppression and PHRR reduction while TNT alone was found inefficient in both tests. Interestingly, when the two nanoparticles are coupled together in a QL system the best results are achieved. Table 10 reports the detailed flammability and cone calorimetry results.

As noticeable, when comparing the data in Table 10 with that in Table 7 and Table 8, the use of polymer/nanoparticle coatings seems be less efficient than an all nanoparticle coating. Indeed, these latter systems not only allow for melt dripping suppression and self-extinguishing behaviour in flammability tests but also strongly increase the TTI in cone calorimetry tests. In particular, the use of Chi appears detrimental in terms of burning rate increase and TTI anticipation.

**Figure 11:**
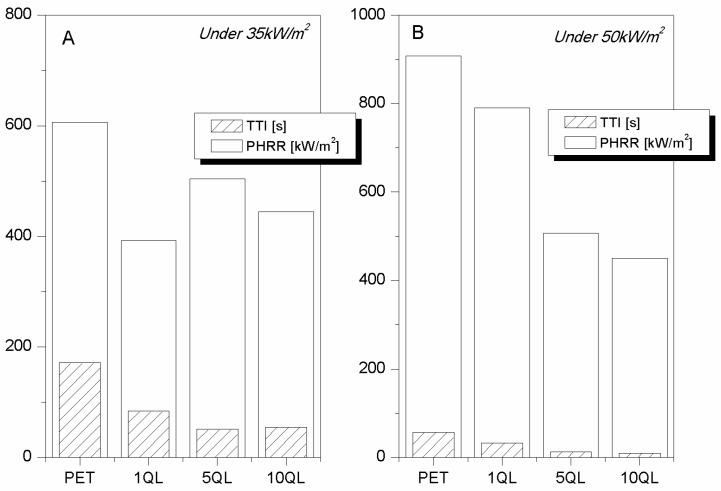
TTI and PHRR values of PET fabrics treated with 1, 5 and 10 QL of char-former assemblies at 35 and 50 kW/m^2^ (**A**,**B**, respectively) [29].

As already observed for the single-step adsorption, the natural evolution in the design of new LbL architectures was a char-former assembly, also described as intumescent-like [28], addressed to promote char formation on the fabric surface due to its constituents (namely, PAA and APP). Thus, more complex architectures consisting of QL repetitive units were deposited on PET fabrics (density: 171 g/m^2^) with add-ons of 3, 13, and 33 wt % for 1, 5, and 10 QL, respectively. The collected data are reported in Table 11 and plotted in Figure 11. Regardless of the heat flux adopted during cone tests, the strong reduction of TTI immediately appears. This result is not surprising as generally a flame retardant system promoting char formation shows this effect; indeed, such a phenomenon occurs at low temperatures and favours polymer decomposition rather than char formation, thus resulting in the production of a protective barrier and less volatile and combustible materials [44]. The action of such systems is clear when observing PET PHRR reductions. At 35 kW/m^2^, the most efficient assembly seems to be 1 QL whereas at 50 kW/m^2^ it is 10 QL (Figure 11). A reasonable explanation of such a trend can be guessed taking into account that the higher the heat flux, the more effective the barrier should be. Thus, a single QL is not able to protect PET at 50 kW/m^2^; conversely, 10 QL is capable of creating a so coherent and consistent coating, able not only to protect fabric from such heat flux, but also from a methane flame application [29]. Table 12 reports the collected data of flammability tests in horizontal and vertical flame spread tests.

In order to integrate the intumescent concept with the heat shielding ability of inorganic nanoparticles, a hybrid LbL coating made from polyallylamine (PAH), sodium polyphosphate (PSP) and TiO_2_ nanoparticles was designed [47]. The coating, deposited repeating a QL repetitive unit (PAH/PSP/PAH/TiO_2_ at 5, 10, and 15 QL) was not able to efficiently improve the PET FR properties as evaluated by PCFC. Indeed, while the unmodified PET yielded a PHRR of 421 w/g, slightly lower values of 380, 371, and 361 w/g were registered for 5, 10, and 15 QL, respectively. Considering the high number of QL employed in the study and comparing the results with that reported in Table 11 and Table 12 for a complete intumescent QL system, it seems that the implementation of TiO_2_ nanoparticles has no beneficial effects on the FR properties achieved. Results may change by varying the coating composition or nanoparticle shape.

#### 2.2.2. Polyester-Cotton Blends

**Figure 12:**
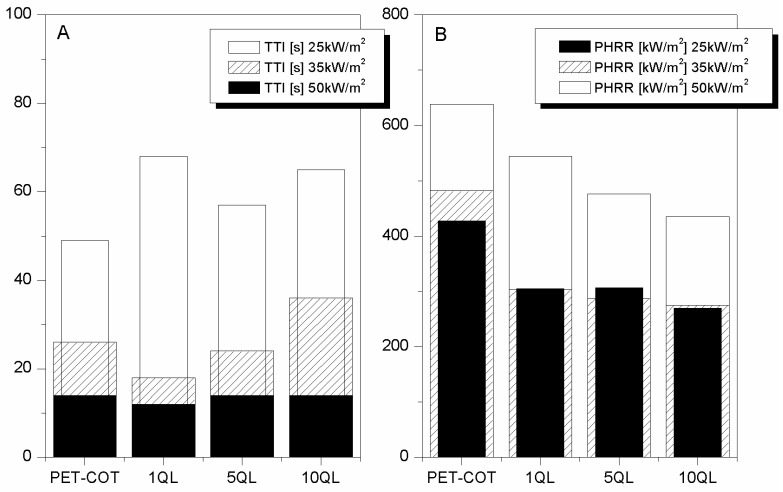
TTI (**A**) and PHRR (**B**) values of PET-COT fabrics treated with 1, 5 and 10 QL at 25, 35, and 50 kW/m^2^ [29].

The quadlayer architectures turned out to be effective also in the case of cotton and for this reason were tested for PET-cotton blend (28 wt % PET) under three different heat fluxes (namely, 25, 35 and 50 kW/m^2^) [29]. The add-ons were 10, 21 and 38 wt % for 1, 5, and 10 QL, respectively. Under the lowest heat flux, the effect on TTI observed for PET was not found here. It is likely that under these heating conditions the QL have time to evolve immediately forming the char barrier able to protect the surrounded substrate. By this way, the so-treated blend can ignite after a longer time period than the untreated one (see Table 13 and Figure 12A), reaching lower PHRR values (Figure 12B). By increasing the heat flux to 35 kw/m^2^, all assemblies turned out to be ineffective to postpone blend TTI (taking into account the experimental error), but efficient enough to further reduce its PHRR by 40%. The same trend was found under the highest heat flux; in this case, only 5 QL and 10 QL are able to significantly reduce blend PHRR in agreement with what was observed for PET fabrics.

In addition, such architectures not only protect a blend from a radiating heat flux but also from a methane flame, similar to what was observed in the case of both PET and cotton [29]. Table 14 shows the collected data for flammability tests in the horizontal and vertical flame spread tests.

Pursuing the deposition of coatings with intumescent features, structures consisting of less complex architectures (BL-based) were designed and compared with systems containing ceramic nanoparticles of silica. More specifically, 5, 10, and 20 BL of chitosan coupled with APP were compared with homologous containing silica instead of chitosan [30]. The Chi/APP pair represents an intumescent system, in which chitosan acts as both carbon source and foaming agent, whereas APP is a thermal promoter in the phosphoric acid release at high temperatures. The latter species can favour the char formation from cellulose contained in cotton. On the other hand, the silica-APP system exploits the synergism between H_3_PO_4_ generated by APP in situ that induces polymer carbonization, and the thermal insulating features of a ceramic like silica.

**Figure 13:**
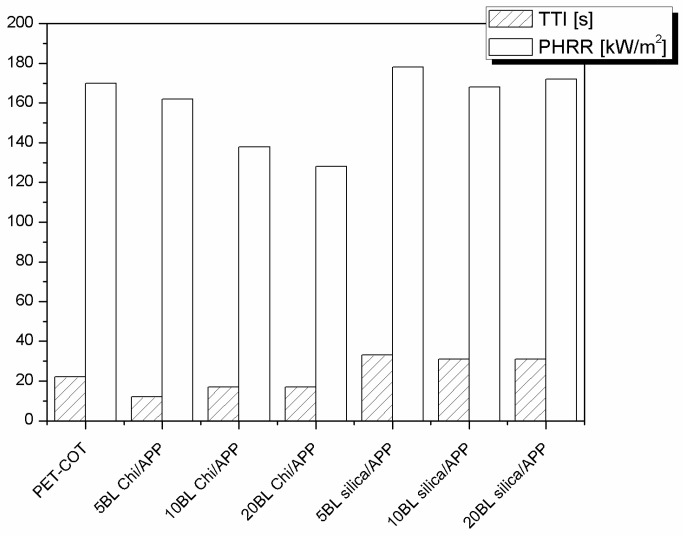
TTI and PHRR values of PET fabrics treated with 10 and 20 BL of Chi/APP or silica/APP assemblies at 35 kW/m^2^ [30]. Data obtained by cone calorimetry at 35 kW/m^2^.

Table 15 summarises the collected data by cone calorimetry which are then plotted in Figure 13. First of all, blend TTI is reduced by chitosan in the treated fabrics, similar to what is already observed in thermogravimetry, due to the -OH groups, which can catalyse the combustion of the cotton. In spite of this, the Chi/APP pair is able to significantly reduce the total heat release and the corresponding rate (PHRR) in a remarkable way (162, 138, and 128 kW/m^2^ for 5, 10, and 20 BL vs. 170 kW/m^2^ untreated blend, respectively). In conclusions, the higher the BL number, the lower is the combustion rate. Furthermore, Chi/APP has proven to be an efficient FR for polyester–cotton blend because favours the char formation, slowing down the production of combustible gases, and promoting the formation of a coherent and consistent final residue, not only after cone calorimetry measurements, but also after vertical flame spread tests (see Table 16).

On the other hand, Silica/APP systems turned out completely ineffective for protecting PET-COT blend, but morphological observation by SEM revealed an analogous problem already observed when depositing silica through NP adsorption. Once again, the procedure was not efficient for depositing a continuous nanocoating on fibres and the resulting morphology was not suitable for forming a protective layer. For this reason, new more complex architectures containing the same LbL counterparts were designed [31]. Therefore, chitosan/APP BLs were stacked with silica/silica BL (i.e., 5 + 5 BL or 10 + 10 BL) and compared with silica/silica/chitosan/APP QL (5 or 10 QL) keeping constant the total number of deposited layers. The architecture consisiting of Chi/APP + silica/silica was employed as the former pair promotes the formation of more coherent residues than those left by silica. Furthermore, a significant increase of TTI and a THR reduction have been achieved by silica/silica pair [25].

Table 17 shows that the proposed architectures are still inefficient for protecting PET-COT blend from a radiating heat flux in spite of the presuppositions. The reason for such behaviour was found in the morphology of the covered fibres assessed by SEM. Unfortunately both types of coatings are stiff and numerous cracks appears on their surface after drying. These cracks represent preferential channels through which the combustible volatile species generated by blend decomposition can escape and promote the combustion. In spite of this, the proposed architectures turned out to be efficient to protect the blend from the application of a methane flame, suppressing the dangerous afterglow phenomenon typical of the untreated substrate (see Table 18).

Recently, a new coating assembly containing chitosan, melamine phosphate and sodium hexametaphosphate was proposed [48]. In the coating concept, melamine is added to the chitosan solution and used as cationic bath while a phosphate solution is used as anionic counterpart. When performing the LbL assembly the formation of a chitosan based coating containing melamine phosphate is reported. In this work different chitosan to melamine ratios were considered, the LbL-treated fabrics were tested by vertical flame spread tests and PCFC; Table 19 reports the main collected data.

As reported in Table 19, only 15 BL deposited with 0.5/0.9 *w*/*w* as Chi/Mel weight ratio allows for self-extinguishment in flammability tests and substantial reductions in PHRR values. The coating was suggested to act in both condensed and gas phases: the improved char formation due to the chitosan/phosphate barrier effect is coupled with the release of ammonia from melamine in the gas phase. With respect to the other chitosan containing coatings described above, the inclusion of melamine appears to result in the best FR performances although achieved after a relatively high number of deposition steps (i.e., 30 layers).

### 2.3. Single-Step Adsorption of Polyelectrolyte Complexes

An alternative approach that somehow couples the potential of the single-step with the versatility of the multi-step adsorption has been recently developed. In this approach the constituents that would normally be employed in a LbL coating are mixed together, under specific conditions (e.g., concentration, pH, ionic strength), in order to form a stable polyelectrolyte complex (PECs) suspension [49]. Then, the fabric is dipped in the complex suspension in a one-step deposition process. On one hand, this approach can be considered relatively simple and fast as a single-step adsorption; on the other hand, it allows for the exploitation of the interactions and intimate mixture between the selected components in the complexion phase typical of a LbL assembled coating.

The described approach was used by Haile et al. for the deposition of complexes made of poly(allylamine hydrochloride) and sodium hexametaphosphate [50]. The same components were also used for the build-up of a LbL coating in order to provide a comparison between the two approaches. A PET-CO fabric treated with PECs (add-on: 17.9 wt %) was capable of self-extinguishing the flame during vertical flame tests. The same result was obtained by the LbL approach only after the deposition of 30 BL. This demonstrates that the use of PECs can be extremely efficient in reducing the number of deposition steps required to obtain substantial FR improvements. Furthermore, the coating durability was also evaluated. PEC treated fabrics subjected to a post treatment in acidic buffer were able to maintain the FR properties after five detergent washings or after 8 h in boiling water.

## 3. Conclusions and Perspectives

When thinking about a new scientific technique that is capable of literally outperforming the performances of the currently standard solutions, the question of a possible projection towards real industrialization arises almost immediately. The answer to this question, which is obviously related to timing and effort, is crucial in order to determine the real societal impact of the proposed approach.

The same question can be, and has been, formulated for the NP adsorption and LbL assembly of flame retardant coatings. The answer is inevitably complex. As reviewed in this paper, recent inputs from the scientific literature have proven that it is possible to minimize the number of deposition steps required, adopt new fast deposition tools such as spraying and obtain durable coatings. For the above reasons, the path appears to be well established thus the targets for the near future can be summarized as follows:

*Improve processing conditions*: one of the main critics often attributed to LbL is represented by the required high number of depositions steps and the excessively long deposition times. Here we have presented results from some papers that are capable of overcoming this problem by either using spray deposition techniques or the newly proposed polyelectrolyte complexes approach.

*Confer the durability and anti-ageing performances to coatings*: durability of LbL coatings on fabrics is mandatory as well as the detailed understanding of their ageing properties. Recent preliminary works have demonstrated that LbL coatings possess an inherent durability that can be further improved by post cross-linking processes.

*Use of non-toxic constituents for the coating*: some of the current flame retardant chemicals are under scrutiny because of their toxicity and environmental problems. This has been brought up after years of specific studies involving different research fields. As a result, the scientific and industrial community have become more susceptible, aware, and cautious of this topic. Thus, a proper evaluation of the toxicological aspect of the deposited LbL coatings (especially the ones containing nanoparticles) is mandatory before planning any industrialization step.

*Develop a practical way for LbL deposition*: this can be considered as the last step for industrialization provided that the challenges related to (i) efficiency; (ii) durability, and (iii) non-toxicity have been successfully addressed.

## Figures and Tables

**Table 1:** Combustion data of untreated and nanoparticle (NP)-treated polyethylene terephthalate (PET) by cone calorimetry (35 kW/m^2^) [22,32,33].

Sample	TTI ± σ [s]	THR ± σ [MJ/m^2^]	EHC ± σ [MJ/kg]	PHRR ± σ [kW/m^2^]	FPI * ± σ [s·m^2^/kW]	[CO] yield ± σ [Kg/Kg]	[CO_2_] yield ± σ [Kg/Kg]
PET (171 g/m^2^)	160 ± 4	2.5 ± 0.6	21.8 ± 7.8	90 ± 9	1.89 ± 0.05	0.0486 ± 0.0228	3.85 ± 1.68
PET_CNa	192 ± 7	2.8 ± 0.4	17.5 ± 2.5	80 ± 2	2.35 ± 0.04	0.0409 ± 0.0008	3.22 ± 0.14
PET_HT	226 ± 4	2.5 ± 0.9	16.4 ± 1.5	76 ± 7	2.97 ± 0.05	0.0261 ± 0.0011	2.43 ± 0.07
PET_OS1	120 ± 6	2.4 ± 0.2	15.0 ± 0.9	70 ± 6	1.50 ± 0.06	0.0301 ± 0.0014	2.73 ± 0.06
PET_TiO_2_	152 ± 6	2.3 ± 0.5	19.4 ± 5.0	70 ± 1	2.17 ± 0.33	0.0421 ± 0.0170	3.58 ± 1.19
PET_SiO_2_	105 ± 9	2.7 ± 0.1	16.0 ± 0.2	81 ± 11	1.30 ± 0.11	0.0302 ± 0.0006	2.71 ± 0.12
PET_POSS	130 ± 9	2.4 ± 0	20.0 ± 6.3	66 ± 3	1.97 ± 0.06	0.0415 ± 0.0158	3.04 ± 0.93
PET_HTSiO_2_	240 ± 8	3.1 ± 0.9	17.0 ± 2.0	85 ± 8	2.82 ± 0.06	0.0123 ± 0.0006	2.81 ± 0.14

* Calculated as TTI/PHRR ratio, as reported in ref. [34].

**Table 2:** Combustion data of PET treated previously by plasma and sodium cloisite by cone calorimetry (35 kW/m^2^) [35].

Sample	TTI ± σ [s]	THR ± σ [MJ/m^2^]	PHRR ± σ [kW/m^2^]	FPI ± σ [s·m^2^/kW]
PET (171 g/m^2^)	158 ± 4	2.5 ± 0.4	90 ± 9	1.76 ± 0.06
PET_CNa	198 ± 8	2.8 ± 0.4	105 ± 2	1.89 ± 0.03
PET_CNa_120 */60 **	261 ± 10	2.6 ± 0.1	89 ± 7	2.93 ± 0.03
PET_CNa_120 */80 **	192 ± 34	2.3 ± 0.4	94 ± 12	2.04 ± 0.15
PET_CNa_120 */15 **	244 ± 24	2.4 ± 0.4	85 ± 7	2.87 ± 0.08
PET_CNa_50 */180 **	241 ± 10	2.2 ± 0.8	92 ± 19	2.62 ± 0.07
PET_CNa_80 */180 **	322 ± 29	1.8 ± 0.4	81 ± 18	3.98 ± 0.16

* Plasma power [w]; ** Plasma time [s].

**Table 3:** Coating composition and resistance to a 100 mm flame penetration applied for 12 min on non-woven PET fabrics treated by starch/SPB coatings [37].

Starch [wt %]	SPB [wt %]	Weight gain [%]	Time to penetration [s]
0	0	0	10
1.0	22.9	42.5	700
3.0	22.9	48.4	NO PENETRATION
5.0	22.9	52.3	110
0.4	21.1	34.3	410
0.8	19.5	33.2	170
1.1	17.7	18.8	150
1.1	17.7	21.8	210
2.3	12.2	22.2	150
3.2	8.3	8.6	20
4.5	2.3	9.9	20

**Table 4:** Coating composition and flame retardant (FR) performances of PET fabrics treated with waterborne polyurethane (PU)-FR coatings [39].

Coating components	Horizontal flame test	UL94 rating	LOI [%]
Untreated PET	Melt dripping	N.C. *	18.5
PU/Fyrol-6 + OP550	No melt dripping, self-extinguishment	V0	25.2
PU/ODDP + OP550			26.1
PU/Fyrol-6/ ODDP + OP550			26.9

* Not Classifiable.

**Table 5:** Combustion data of untreated and NP-treated PET-COT_85:15 by cone calorimetry (35 kW/m^2^) [22].

Sample	TTI ± σ [s]	THR ± σ [MJ/m^2^]	PHRR ± σ [kW/m^2^]	FPI ± σ [s·m^2^/kW]	[CO] yield ± σ [Kg/Kg]	[CO_2_] yield ± σ [Kg/Kg]
PET-COT_85:15 (280 g/m^2^)	35 ± 5	3.7 ± 0.1	150 ± 1	0.24 ± 0.07	0.0380 ± 0	2.94 ± 0.03
PET-COT_85:15_CNa	52 ± 5	3.6 ± 0.6	107 ± 13	0.49 ± 0.19	0.0370 ± 0.0034	2.62 ± 0.20
PET-COT_85:15_SiO_2_	56 ± 7	3.9 ± 0.3	128 ± 8	0.44 ± 0.09	0.0316 ± 0.0015	2.55 ± 0.07

**Table 6:** Combustion data of untreated and NP-treated PET-COT_65:35 by cone calorimetry (35 kW/m^2^) [22].

Sample	TTI ± σ [s]	THR ± σ [MJ/m^2^]	PHRR ± σ [kW/m^2^]	FPI ± σ [s·m^2^/kW]	[CO] yield ± σ [Kg/Kg]	[CO_2_] yield ± σ [Kg/Kg]
PET-COT_65:35 (245 g/m^2^)	11 ± 1	3.4 ± 0.2	140 ± 5	0.08 ± 0.06	0.0313 ± 0.0021	2.91 ± 0.03
PET-COT_65:35_CNa	24 ± 4	3.7 ± 0.1	125 ± 7	0.19 ± 0.13	0.0328 ± 0.0049	2.54 ± 0.11
PET-COT_65:35_SiO_2_	20 ± 1	3.6 ± 0.1	130 ± 5	0.15 ± 0.04	0.0265 ± 0.0028	2.57 ± 0.21
PET-COT_65:35_HT	74 ± 1	2.7 ± 0.3	121 ± 1	0.61 ± 0.01	0.0304 ± 0.0023	2.71 ± 0.54
PET-COT_65:35_HTSiO_2_	54 ± 1	2.6 ± 0.3	115 ± 4	0.47 ± 0.03	0.0281 ± 0.0041	2.69 ± 0.47

**Table 7:** Combustion data of untreated and BL-treated PET by cone calorimetry (35 kW/m^2^).

Sample	TTI ± σ [s]	PHRR ± σ [kW/m^2^]	ΔPHRR [%]	FPI ± σ [sm^2^/kW]	Reference
PET (171 g/m^2^)	222 ± 3	54 ± 4		4.11 ± 0.04	[25]
5 BL	321 ± 31	44 ± 1	−19	7.30 ± 0.06	
10 BL	254 ± 32	51 ± 2	−6	4.98 ± 0.08	
20 BL	212 ± 3	52 ± 5	−4	4.08 ± 0.06	
PET (490 g/m^2^)	84 ± 4	303 ± 15		0.28 ± 0.05	[26]
Dipping	96 ± 5	293 ± 15	−3	0.33 ± 0.05	
Horizontal spray	102 ± 5	200 ± 10	−34	0.51 ± 0.05	
PET (490 g/m^2^)	93 ± 4	340 ± 8		0.27 ± 0.03	[27]
5 BL ZrP/PDAC	157 ± 2	350 ± 1	3	0.45 ± 0.01	
10 BL ZrP/PDAC	173 ± 8	306 ± 13	−10	0.57 ± 0.04	
5 BL ZrP/ POSS	107 ± 5	252 ± 12	−26	0.42 ± 0.05	
10 BL ZrP/POSS	104 ± 7	273 ± 9	−20	0.38 ± 0.05	
5 BL ZrP/silica	159 ± 20	286 ± 27	−16	0.56 ± 0.11	
10 BL ZrP/silica	135 ± 15	298 ± 12	−12	0.45 ± 0.08	

**Table 8:** Combustion data of untreated and LbL-treated PET by flame spread tests according to the ASTM D6413 standard [25].

Sample	Burning time [s]	Afterflame time [s]	Dripping	Weight loss [%]
PET (171 g/m^2^)	32	20	YES	8
5 BL	10	-	NO	1
10 BL	2	-	NO	-
20 BL	10	-	YES	2

**Table 9:** PCFC data and durability performances for PET treated by BPEI/CNa LbL coating [45].

Sample	PHRR ± σ [W/g]	THR ± σ [kJ/g]	Char yield ± σ [%]	Durability
PET (174 g/m^2^)	629 ± 32	25.3 ± 0.79	12.1 ± 0.6	-
10 BL BPEI/CNa	393 ± 10	24.6 ± 0.35	16.0 ± 0.3	Durable to 5 washing cycles with deionized water and hexane
20 BL BPEI/CNa	368 ± 15	23.9 ± 0.34	16.7 ± 0.9	

**Table 10:** Flammability and cone calorimetry (under 35 kW/m^2^) data for PET treated by CNa- and TNT-based LbL coatings [46].

Sample		Horizontal flame spread test		Cone calorimetry			
	Add-on [%]	Burning rate [mm/s]	Dripping	TTI ± σ [s]	PHRR ± σ [kW/m^2^]	THR ± σ [MJ/m^2^]	Char yield ± σ [%]
PET (170 g/m^2^)	-	1.5	YES	25 ± 3	117 ± 5	1.1 ± 0.1	27 ± 2
4 BL Chi/CNa	2.1	Not assessed		17 ± 2	90 ± 6	1.0 ± 0.3	31 ± 1
8 BL Chi/CNa	3	1.9	NO	15 ± 2	73 ± 7	0.9 ± 0.2	34 ± 3
4 BL Chi/TNT	5.1	Not assessed		17 ± 3	99 ± 9	1.0 ± 0.5	27 ± 3
8 BL Chi/TNT	7.2	1.6	YES	14 ± 4	84 ± 6	1.0 ± 0.2	30 ± 2
2 QL Chi/CNa/ Chi/TNT	2	Not assessed		16 ± 2	77 ± 5	0.8 ± 0.1	35 ± 2
4 QL Chi/CNa/ Chi/TNT	4.1	2.0	NO	13 ± 2	61 ± 6	0.7 ± 0.3	42 ± 1

**Table 11:** Combustion data of untreated and QL-treated PET by cone calorimetry under different heat fluxes [29].

Sample	TTI ± σ [s]	PHRR ± σ [kW/kg]	ΔPHRR [%]
	*Heat flux* = 35 kW/m^2^		
PET (171 g/m^2^)	171 ± 27	606 ± 37	-
1 QL	84 ± 9	392 ± 9	−35
5 QL	51 ± 5	504 ± 23	−17
10 QL	55 ± 11	444 ± 20	−27
	*Heat flux* = 50 kW/m^2^		
PET	57 ± 3	908 ± 31	-
1 QL	33 ± 3	791 ± 8	−13
5 QL	13 ± 1	507 ± 16	−44
10 QL	10 ± 1	451 ± 7	−50

**Table 12:** Combustion data of untreated and QL-treated PET by horizontal and vertical flame spread tests [29].

Sample	Burning time [s]	Dripping	Residue [%]
Horizontal configuration			
PET (171 g/m^2^)	78	YES	42
1 QL	8	YES	98
5 QL	8	NO	95
10 QL	7	NO	94
Vertical configuration			
PET (171 g/m^2^)	14	YES	73
1 QL	8	YES	84
5 QL	21	NO	20
10 QL	22	NO	35

**Table 13:** Combustion data of untreated and QL-treated PET-COT by cone calorimetry at different heat fluxes [29].

Sample	TTI ± σ [s]	PHRR ± σ [kW/kg]	ΔPHRR [%]
	*Heat flux = 25 kW/m^2^*		
PET-COT (340 g/m^2^)	49 ± 8	427 ± 20	-
1 QL	68 ± 9	305 ± 23	−29
5 QL	57 ± 6	307 ± 4	−28
10 QL	65 ± 10	270 ± 13	−37
	*Heat flux* = 35 kW/m^2^		
PET-COT	26 ± 5	482 ± 23	-
1 QL	18 ± 2	303 ± 5	−37
5 QL	24 ± 4	287 ± 12	−40
10 QL	36 ± 14	275 ± 64	−43
	*Heat flux* = 50 kW/m^2^		
PET-COT	14 ± 2	638 ± 41	-
1 QL	12 ± 1	544 ± 24	−15
5 QL	14 ± 1	476 ± 10	−25
10 QL	14 ± 2	435 ± 26	−32

**Table 14:** Combustion data of untreated and QL-treated PET-COT fabrics by horizontal and vertical flame spread tests [29].

Sample	Burning time [s]	After-flame time [s]	Residue [%]
Horizontal configuration			
PET-COT (340 g/m^2^)	210	35	0
1 QL	270	-	12
5 QL	14	-	96
10 QL	6	-	97
Vertical configuration			
PET-COT	39	95	0
1 QL	40	-	7
5 QL	34	-	31
10 QL	22	-	56

**Table 15:** Combustion data of untreated and BL-treated PET-COT by cone calorimetry (35 kW/m^2^) [30].

Sample	TTI ± σ [s]	PHRR ± σ [kW/m^2^]	ΔPHRR [%]
PET-COT (340 g/m^2^)	22 ± 2	170 ± 3	-
5 BL Chi/APP	12 ± 3	162 ± 6	−5
10 BL Chi/APP	17 ± 6	138 ± 16	−19
20 BL Chi/APP	17 ± 4	128 ± 3	−25
5 BL Silica/APP	33 ± 12	178 ± 10	5
10 BL Silica/APP	31 ± 5	168 ± 8	−1
20 BL Silica/APP	31 ± 9	172 ± 9	+1

**Table 16:** Combustion data of untreated and BL-treated PET-COT fabrics by vertical flame spread tests [30].

Sample	Burning time [s]	After-flame time [s]	Residue [%]
PET-COT (340 g/m^2^)	35	36	0
5 BL Chi/APP	35	-	4.5
10 BL Chi/APP	34	-	5.8
20 BL Chi/APP	34	-	7.1
5 BL Silica/APP	39	-	2.5
10 BL Silica/APP	38	-	3.8
20 BL Silica/APP	40	-	7.1

**Table 17:** Combustion data of untreated and LbL-treated PET-COT by cone calorimetry (35 kW/m^2^) [31].

Sample	TTI ± σ [s]	PHRR ± σ [kW/m^2^]	ΔPHRR [%]
PET-COT (340 g/m^2^)	22 ± 2	170 ± 3	-
5 + 5 BL	23 ± 1	182 ± 4	+7
10 + 10 BL	12 ± 3	177 ± 9	+4
5 QL	26 ± 2	163 ± 6	−4
10 QL	35 ± 1	151 ± 7	−11

**Table 18:** Combustion data of untreated and LbL-treated PET-COT fabrics by vertical flame spread tests [31].

Sample	Burning time [s]	After-flame time [s]	Residue [%]
PET-COT (340 g/m^2^)	35	36	0
5 + 5 BL	40	-	3.0
10 + 10 BL	34	-	5.5
5 QL	39	-	1.7
10 QL	37	-	3.8

**Table 19:** Flammability and PCFC data for PET-COT (107 g/m^2^) treated by chitosan, melamine phosphate LbL coatings. All coatings were deposited in order to achieve a constant 12 wt % loading [48].

Chi/Mel [w/w]	Burning time ± σ [mm/s]	Self-extinguishing	Char yield ± σ [%]	PHRR ± σ [W/g]	THR ± σ [kJ/g]
PET-COT (107 g/m^2^)	12 ± 2	NO	49 ± 1	127 at 387 °C and 242 at 463 °C	15.2 ± 0.2
1.4/0 (8 BL)	26 ± 2	NO	28 ± 2	55 at 311 °C and 199 at 457 °C	10.9 ± 0.2
0.9/0.5 (10 BL)	33 ± 2	NO	31 ± 1	27 at 318 °C and 147 at 428 °C	9.4 ± 0.1
0.5/0.9 (15 BL)	0	YES	93 ± 1	25 at 322 °C and 171 at 418 °C	9.6 ± 0.1

## Abbreviations

The following abbreviations are used in this manuscript:

APP	ammonium polyphosphate
BL	bilayer
BPEI	branched polyethylenimine
Chi	chitosan
CNa	sodium cloisite
EHC	effective of heat combustion
FPI	fire performance index
FR	flame retardant
HRR	heat release rate
HT	hydrotalcite
LbL	Layer by Layer
LOI	limiting oxygen index
NP	nanoparticle
ODDP	octahydro-2,7-di(N,N-dimethylamino)-1,6,3,8,2,7 dioxadiazadiphosphecine
OS	bohemite
PAH	polyallylamine
PCFC	pyrolysis-combustion flow calorimetry
PDAC	polydiallyldimethylammonium chloride
PEC	polyelectrolyte complex
PET	polyethylene terephthalate
PET-COT	polyester-cotton
PHRR	heat release rate peak
POSS^®^	polyhedral oligomeric silsesquioxane
PSP	sodium polyphosphate
PU	polyurethane
QL	quadlayer
SEM	scanning electron microscopy
SPB	sodium polyborate
TNT	titanate nanotube
TTI	time to ignition
ZrP	zirconium phosphate
